# Solasonine Inhibits Pancreatic Cancer Progression With Involvement of Ferroptosis Induction

**DOI:** 10.3389/fonc.2022.834729

**Published:** 2022-04-12

**Authors:** Xiaoqiang Liang, Cheng Hu, Mian Han, Congying Liu, Xun Sun, Kui Yu, Honggang Gu, Jingzhe Zhang

**Affiliations:** ^1^Institute of Chinese Traditional Surgery, Longhua Hospital Affiliated to Shanghai University of Traditional Chinese Medicine, Shanghai, China; ^2^Experiment Center for Science and Technology, Shanghai University of Traditional Chinese Medicine, Shanghai, China; ^3^Gastrointestinal surgery, Longhua Hospital Affiliated to Shanghai University of Traditional Chinese Medicine, Shanghai, China; ^4^General surgery, Longhua Hospital Affiliated to Shanghai University of Traditional Chinese Medicine, Shanghai, China; ^5^Hepatobiliary surgery, Longhua Hospital Affiliated to Shanghai University of Traditional Chinese Medicine, Shanghai, China

**Keywords:** solasonine, pancreatic cancer, ferroptosis, SLC7A11, OTUB1, TFAP2A

## Abstract

Pancreatic cancer is a highly fatal malignant tumor of the digestive system. It is characterized by early metastasis and high mortality rates. Solasonine, a steroidal alkaloid, is derived from *Solanum nigrum L.*, a natural herb. Solasonine is associated with excellent anti-tumor effects, however, its effects on pancreatic cancer have not been fully established. Pancreatic cancer cells (PANC-1 and CFPAC-1) were used to verify the *in vitro* and *in vivo* effects of solasonine. Metabolomics were used to evaluate its underlying mechanisms. Solasonine promoted PANC-1 and CFPAC-1 cell apoptosis while inhibiting their proliferation, migration and invasion. Mouse xenograft models and metastasis models of ANC-1 and CFPAC-1 confirmed that solasonine blocked tumor formation and metastasis. Metabolomics confirmed the effects of solasonine on glutathione metabolism and SLC7A11-mediated ferroptosis. Furthermore, Co-Immunoprecipitation and Duolink^®^ *in situ* PLA confirmed that OTUB1, a deubiquitylating enzyme, interacted with SLC7A11 and solasonine to enhance ubiquitinated degradation of SLC7A11 in PANC-1 and CFPAC-1 cells. Besides, molecular docking confirmed that solasonine directly bound TFAP2A and suppressed its protein levels. Bioinformatics and luciferase assays revealed that TFAP2A binds the OTUB1 promoter region, thereby promoting its transcription. In summary, solasonine inhibits the TFAP2A/OTUB1 SLC7A11 axis to activate ferroptosis and suppress pancreatic cancer cell progression.

## Introduction

Globally, pancreatic cancer (PC) is a highly fatal malignancy of the digestive system and is the seventh most common cause of cancer-related mortality among men and women (1). According to GLOBOCAN 2020 estimates, there were approximately 495,773 new cases and approximately 466,003 mortalities from pancreatic cancer. It has been postulated that pancreatic cancer will overtake breast cancer as the third leading cause of cancer-related deaths in the European Union. Cancer statistics from the US Surveillance, Epidemiology, and End Results (SEER) database from 2010 to 2016 estimates that in 2020, there will be 57,600 new cases of pancreatic cancer and 47,050 mortalities from pancreatic cancer (2). By the year 2030, pancreatic cancer is expected to be the second leading cause of cancer-related deaths in the United States (3). Based on the 2000 - 2011 data from the National Cancer Centre of China, pancreatic cancer-related incidences and mortality rates are on the rise (4). The 2015 malignancy registry data in China indicates that there were about 95,000 new cases of pancreatic cancer and about 85,000 mortalities from pancreatic cancer (5). In China, pancreatic cancer has become the sixth leading cause of cancer-related mortalites^5^. Although there have been advances in diagnostic methods, perioperative management strategies, radiotherapeutic techniques and systemic treatment for advanced disease, due to a lack of early specific symptoms and effective screening tools, most of the pancreatic cancer patients present with metastases at the time of initial diagnosis, with more than half of them exhibiting distant metastases. The 5-year survival rate for this group of patients with distant metastases is less than 3% (2). Globally, pancreatic cancer-associated mortality rate has remained high, and is a major public health challenge and an economic burden.

Ferroptosis is a newly discovered mode of programmed cell death. In contrast to apoptotic and necrotic cell death, ferroptosis is morphologically characterized by mitochondrial wrinkling and narrowing of mitochondrial ridges, accompanied with intracellular-specific lipid peroxidation and iron ion accumulation (6). In 2012, Hirschhorn et al. (7) reported that Erastin can induce a form of cell death in Ras mutant tumor cells that was not characterized by other types of cell death, and that this form of death could not be reversed by inhibitors of apoptosis and necroptosis, however, it could be inhibited by the chelator of iron ions, deferoxamine. They named this form of cell death as ferroptosis. Not only is ferroptosis involved in tumor development, it also plays an important role in various physiopathological processes, such as neurological injury and degenerative diseases (8), acute renal failure (9), drug-induced liver injury (10), cardiac ischemia-reperfusion injury and T-cell immunodeficiency-related diseases (11). Various ferroptosis inducers have been identified, such as salazo sulfa pyrimidine, sorafenib and artesunate. These ferroptosis inducers can specifically induce ferroptosis in various cells (12, 13). Currently, regulatory molecules for ferroptosis are divided into two main categories: those that promote ferroptosis (iron ions, reactive oxygen species, long-chain polyunsaturated fatty acids, p53, and NCOA4 among others) and those that inhibit ferroptosis (reduced glutathione, NRF2, and System Xc- among others) (14, 15). In addition, other related regulators have been identified including GPX4, the core protein of ferroptosis, HSPA5, which is involved in oxidative stress, ACSL4, which is associated with lipid synthesis, and transferrin, which is related to ferric ions (16, 17). In the presence of ferroptosis inducers, intracellular iron storage proteins are degraded by iron autophagy thereby releasing iron ions into the cytoplasm as free iron ions, which then generate reactive oxygen species through the Fenton reaction. The free reactive oxygen species oxidizes normal lipids in the cell membrane or cell wall to lipid peroxide, which accumulates in excess, leading to ferroptosis (18, 19). System Xc-, which is located in the cell membrane, transports glutamate out of the cell and used as a raw material for the synthesis of reduced glutathione. Glutathione peroxidase 4 (GPX4) uses reduced glutathione as a substrate to reduce oxidized lipids to normal lipids, preventing the accumulation of peroxides, and thus inhibiting ferroptosis (20). Therefore, the promoters and inhibitors of ferroptosis influence the occurrence of ferroptosis by altering the accumulation of lipid peroxides (21).

*Solanum nigrum L.*, a dual-purpose plant of the Solanaceae family, is widely distributed throughout the world. There are more than 20 species forming the *Solanum nigrum L.* complex species system, of which four species are mainly distributed in China, namely; *Lobelia, Lobelia olivacea, Lobelia yellow-fruited* and *Lobelia red-fruited* (22). *Solanum nigrum L.* exhibits anti-tumor activities, particularly against breast and bladder cancers (23, 24). It has also been shown to be effective in the treatment of colitis (25), inhibition of liver fibrosis, and exhibits anti-hepatitis virus (26), hepatoprotective (27), antipyretic and analgesic (28), as well as antihypertensive effects. The major active constituents of *Solanum nigrum L.* include: ligand-like alkaloids, polysaccharides, saponins and other nutrients. The alkaloids include solasonine, solamargine and lobeline, among which solasonine and solamargine can be hydrolyzed into solasodine to exert anti-tumor effects. The polysaccharides include water-soluble (SNL-1, SNL-2, PS) and basic polysaccharides (SNL-3, SNL-4, and SNLPS) while saponins include 11 compounds, with degalactoside and lobeline being among them. The nutritional components include sugars, fats, vitamins and amino acids (22). The structure and functions of solasonine have been highly elucidated. Solasonine occurs in three main forms: α-solasonine, β-solasonine and γ-solasonine, of which α-solasonine is the most abundant. In addition to its insecticidal effects, C. M. Lezama-Davila et al. (29) found that the combination of solasonine with solamargine was more effective than the recommended drug (sodium antimony gluconate) in killing intracellular and extracellular Leishmania, and most importantly, the combination exerted significant antitumor effects on various tumor cells. Solasonine is a potential therapeutic agent for tumors. It has significant inhibitory effects on lung cancer, breast cancer, bladder cancer, liver cancer, cervical cancer, gastric cancer and on other tumor cells (30). Moreover, it promotes the apoptosis of gastric cancer cells by elevating the levels of apoptosis proteins (Bax and Caspase-3), and protein levels of the apoptosis inhibitor (BCL-2) (31). The inhibitory effects of solasonine on the hepatocellular carcinoma cell line, HepG2, were shown to be mediated by the induction of apoptosis by upregulating Bax and downregulating Bcl-2 mRNA expressions (32). In addition, solasonine inhibits the proliferation of various hepatocellular carcinoma cell lines through reciprocal regulation of miR-375-3p and IncRNA-CCAT1, which results in the induction of transcription factor SP1. Inhibition of HepG2 cell growth was found to be mediated by IRF5 suppression through interactions between miR-375-3p and IncRNA-CCAT1 (33). Former study showed that the binding of solasodine glycosides on tumor cells may be mediated through the monosaccharide rhamnose, which forms part of solasonine, solamargine and di-glycosides. Furthermore, these results provide evidence that solasodine glycosides selectively destroys tumor cells relative to normal cells *in vivo *(34). solasonine could inhibit the hedgehog pathway *via* acting on Gli and suppress drug resistance (35). *In vivo*, a mouse xenograft model of HepG2 tumor formation confirmed that solasonine inhibits tumor sizes as well as weights and suppresses the migration as well as invasion of hepatocellular carcinoma cells. The effects of solasonine on glutathione metabolism, including GPX4 as well as glutathione synthetase (GSS), and inhibition of GPX4-induced ferroptosis are effective therapeutic strategies for triggering cancer cell death (36). However, the effects of solasonine on pancreatic cancer have not been determined. Therefore, we aimed at investigating the effects of solasonine and its potential mechanisms on pancreatic cancer.

## Methods

### Cells and Treatment

PANC-1 and CFPAC-1 cells were purchased from the American Type Culture Collection (ATCC). For PANC-1, cells were grown in Dulbecco’s Modified Eagles Medium (DMEM) supplemented with 10% FBS and incubated at 37°C in a 5% CO_2_ atmosphere. For CFPAC-1, cells were grown in Iscove’s Modified Dulbeccos Medium (IMDM) with 10% FBS and incubated at 37°C in a 5% CO_2_ atmosphere. Solasonine (98%; Shanghai Yuanye Bio-Technology Co., Ltd, Shanghai, China) was dissolved in dimethyl sulfoxide (DMSO) and stored at -80°C.

### Cell Transfection

OTUB1 and TFAP2A overexpression plasmids [pcDNA3.1-OTUB1 (OTUB1-OE) and pcDNA3.1-TFAP2A (TFAP2A-OE)] were obtained from GeneChem (Shanghai, China). The lipofectamine 2000 (Invitrogen, MA, U.S.) reagent was used to transfect cells with the appropriate amount of vector. Then, cells were incubated for 24 h. Expression efficiencies in cells transfected with OTUB1-OE or TFAP2A-OE were determined using qPCR.

### Animal Studies

For xenograft assays, we subcutaneously injected 1×10^6^ PANC-1 and CFPAC-1 into the right side of each male nude mouse (n=6). Tumor volumes (length × width^2^ × 0.5) were measured at specified time points. For one treatment cycle in a week (starting from week 1 to week 5), solasonine (40 or 80 mg/kg, oral administration, 2 times) were given. A total of five treatment cycles were conducted in this experiment. Five weeks after injection, tumors were excised.

To examine the role of Solasonine in a pancreatic cancer metastasis model, the experiments were performed with 5 mice in each group. A PANC-1 and CFPAC cell line with stable luciferase expression (PANC-1-Luc and CFPAC-1-Luc) was generated. We injected 1×10^6^ PANC-1-Luc and CFPAC-1-Luc intravenously through the tail vein into male nude mice (Chinese Science Academy, Shanghai, China). For one treatment cycle in a week (starting from week 1 to week 6) and Solasonine (40 and 80 mg/kg, oral administration, 2 times) were given. Total six treatment cycles were conducted in this experiment. One month later, we measured and quantified the pancreatic metastases by an *in vivo* bioluminescent imaging with an IVIS Lumina Series III *in vivo* Imaging System (PerkinElmer, New York, NY). Before imaging, 450 μl of 10 mg/ml d-Luciferin dissolved in PBS was intraperitoneally injected into each mouse, and 15 min later, the mice were anesthetized with isoflurane and imaged by the IVIS Imaging System.

All animal experiments were conducted in accordance with recommendations of the Animal Care Committee of Shanghai University of Traditional Chinese Medicine. The protocol was reviewed and approved by the Shanghai University of Traditional Chinese Medicine Institutional Review Board (Permit Number: PZSHUTCM210305012). Surgery was performed under sodium pentobarbital anesthesia with all efforts being made to minimize suffering.

### Statistical Analysis

Statistical analyses were performed using R (R version 3.6.3). A T-test was used to compare two independent groups, while one-way ANOVA was used to evaluate differences among groups. *p* < 0.05 was considered statistically significant.

Details of materials and methods are available in **Supplementary Methods**.

## Results

### Solasonine Inhibited Pancreatic Cancer Cell Progression *In Vitro*

**Figure 1:**
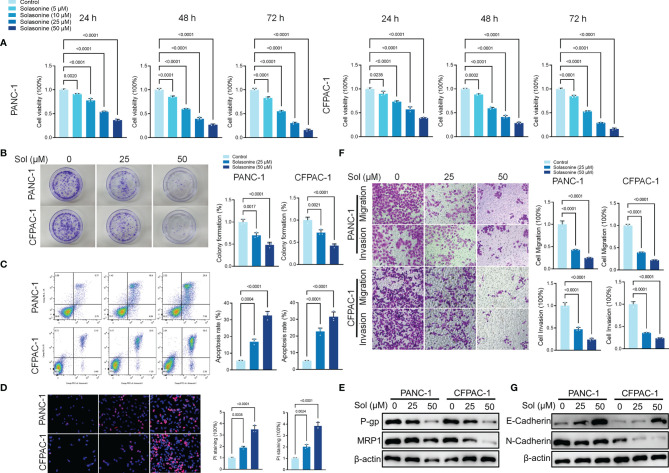
Solasonine inhibited pancreatic cancer cell progression *in vitro.* **(A)** CCK8 results showing the viability of PANC-1 and CFPAC-1 cells after treatment with different concentrations of solasonine for 24, 48, and 72 h. **(B)** Colony formation assay showing proliferations of PANC-1 and CFPAC-1 cells after treatment with solasonine for 24 h. **(C)** Cell apoptosis, as detected by flow cytometry. **(D)** Propidium iodide (PI) staining (red) indicates apoptotic/necrotic cells. **(E)** Western blot demonstrating P-gp and MRP1 levels in PANC-1 and CFPAC-1 cells after treatment with solasonine for 24 h. **(F)** Cell migration and invasion were detected by Transwell assays. **(G)** Western blot demonstrating E-cadherin and N-cadherin protein levels in PANC-1 and CFPAC-1 cells after treatment with solasonine for 24 h. Data are shown as mean ± SD for n=3.

To verify the effects of solasonine on pancreatic cancer, first, we cultured pancreatic cancer cells (PANC-1 and CFPAC-1). Then, the cultured cells were treated with different concentrations of solasonine (5, 10, 25 and 50 μM) for 24, 48 and 72 h. The Cell Counting Kit-8 (CCK-8) assay revealed that solasonine suppressed cell viabilities in a dose dependent manner, with inhibitory effects of solasonine being strongest at 72 h (**Figure 1A**). Similarly, clone formation assays showed that solasonine (25 and 50 μM) prevented PANC-1 and CFPAC-1 cell proliferation (**Figure 1B**).The annexin V-positive cells consisted of propidium iodide–negative (early apoptotic) and propidium iodide–positive (late apoptotic) cells (propidium iodide staining not shown). The flow cytometry (**Figure 1C**) and propidium iodide (PI) staining (**Figure 1D**) revealed that solasonine (25 and 50 μM) for 24 h facilitated cell apoptosis. Besides, levels of drug resistance related proteins (MRP1 and P-gp) were suppressed after treatment with solasonine (**Figure 1E**). To assess the effects of solasonine on cell metastatic abilities, transwell assays were performed to assess cell migration and invasion (**Figure 1F**), while western blot was used to determine Epithelial-Mesenchymal Transition (EMT) related protein levels of E-cadherin and N-cadherin (**Figure 1G**). These results shows that solasonine abrogates cell migration and invasion abilities in PANC-1 and CFPAC-1 cells.

### Solasonine Promoted Pancreatic Cancer Cell Ferroptosis *In Vitro*

**Figure 2:**
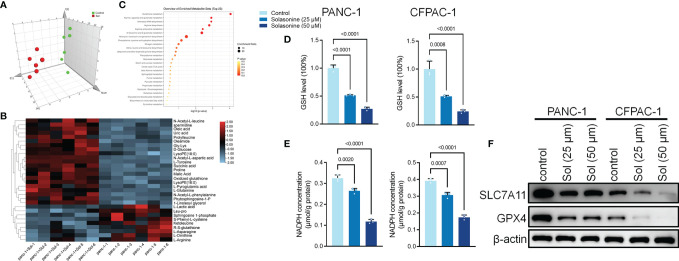
Solasonine regulated Glutathione pathway in pancreatic cancer cells. **(A)** OPLS-DA score plot showing differences between solasonine treatment and control groups in PANC-1 and CFPAC-1 cells. **(B)** A heat map was used to identify metabolites in PANC-1 and CFPAC-1 cells. **(C)** Pathway analysis of significantly altered metabolites in PANC-1 and CFPAC-1 cells, compared to the baseline. **(D)** Cellular GSH levels were detected in PANC-1 and CFPAC-1 cells after treatment with solasonine for 24 h. **(E)** Cellular NADPH levels were detected in PANC-1 and CFPAC-1 cells. **(F)** Western blot demonstrating SLC7A11 and GPX4 levels in PANC-1 and CFPAC-1 cells after treatment with solasonine for 24 h. Data are shown as mean ± SD for n=3.

**Figure 3:**
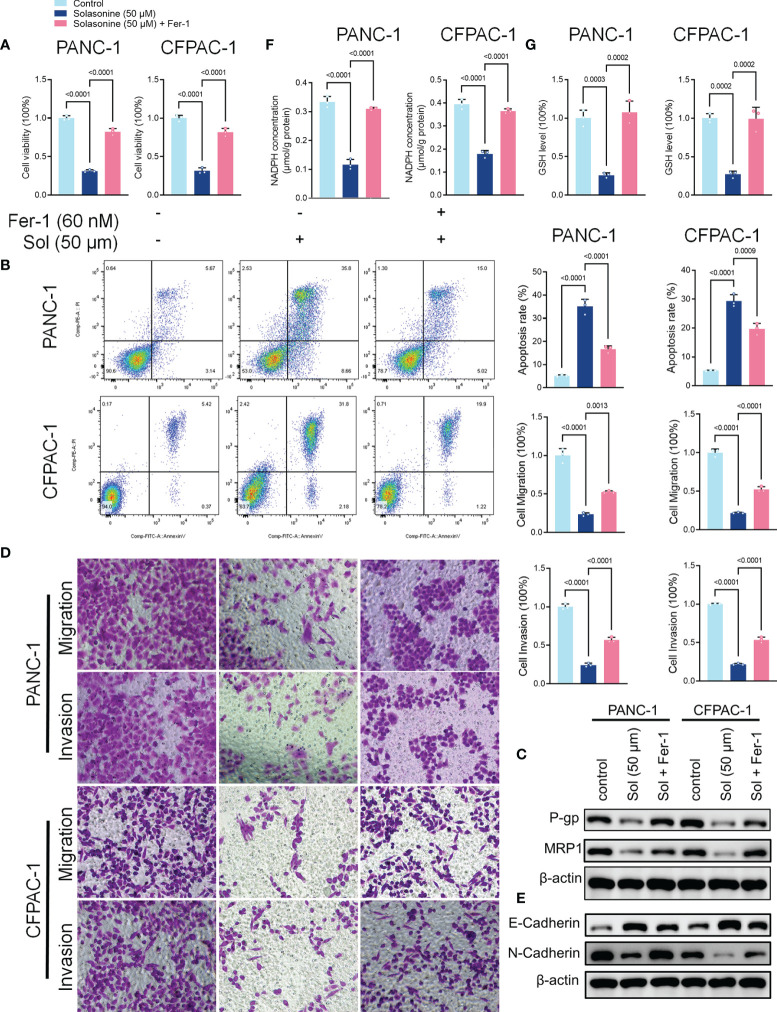
The ferroptosis inhibitor, Ferrostatin-1 (Fer-1), reversed the effects of solasonine in pancreatic cancer cells. **(A)** CCK8 results showing the viability of PANC-1 and CFPAC-1 cells after treatment with solasonine (50 μM) or Fer-1 (20 μM) for 24 h. **(B)** Cell apoptosis as detected by flow cytometry. **(C)** Western blot demonstrating P-gp and MRP1 protein levels in PANC-1 and CFPAC-1 cells after treatment with solasonine (50 μM) or Fer-1 (20 μM) for 24 h. **(D)** Cell migration and invasions were assessed by Transwell assays after treatment with solasonine (50 μM) or Fer-1 (20 μM) for 24 h. **(E)** Western blot demonstrating E-cadherin and N-cadherin levels in PANC-1 and CFPAC-1 cells after treatment with solasonine (50 μM) or Fer-1 (20 μM) for 24 h. **(F)** Cellular GSH levels were detected in PANC-1 and CFPAC-1 cells after treatment with solasonine (50 μM) or Fer-1 (20 μM) for 24 h. **(G)** Cellular NADPH levels in PANC-1 and CFPAC-1 cells after treatment with solasonine (50 μM) or Fer-1 (20 μM) for 24 h. Data are shown as mean ± SD fro n=3.

After incubation with solasonine, we used metabolomics to detect differentially expressed metabolites in PANC-1 cells. The MetaboAnalyst software was used to investigate the underlying alterations in metabolic pathways. As shown in **Figures 2A–C**, solasonine altered the glutathione (GSH) pathway. GSH depletion and lipid peroxidation are significant hallmarks of ferroptosis related cell death. Thus, we evaluated ferroptosis and lipid peroxidation-related indicators, such as glutathione (GSH), nicotinamide adenine dinucleotide phosphate (NADPH), as well as protein levels of ferroptosis markers, SLC7A11 and GPX4. After 24 h of treatment, solasonine (25 and 50 μM) suppressed GSH and NADPH concentrations in PANC-1 and CFPAC-1 cells (**Figures 2D, E**). Besides, solasonine (**Figures 3F, G**) suppressed SLC7A11 and GPX4 protein levels (**Figure 2F**).

To validate the roles of ferroptosis in solasonine inhibition of tumor progression, we treated the cells using the ferroptosis inhibitor, Ferrostatin-1 (Fer-1). As shown in **Figure 3A**, Fer-1 reversed the suppressive effects of solasonine on pancreatic cancer cell proliferation. Besides, pro-apoptotic and anti-drug resistance effects of solasonine on pancreatic cancer cells were suppressed after Fer-1 treatment (**Figures 3B, C**). Transwell and western blot assays confirmed that Fer-1 exacerbated cell metastatic abilities and EMT (**Figures 3D, E**). Fer-1 treatment recovered cell GSH and NADPH concentrations that had been suppressed by solasonine (**Figures 3F, G**). Therefore, we postulated that solasonine inhibits pancreatic cancer cell progression by activating cell ferroptosis, and that ferroptosis inhibitors, such as Fer-1, can reverse the effects of solasonine.

### OTUB1-Mediated Ub/Proteasome Pathway Is Involved in Solasonine-Mediated SLC7A11 Degradation in Pancreatic Cancer Cells

**Figure 4:**
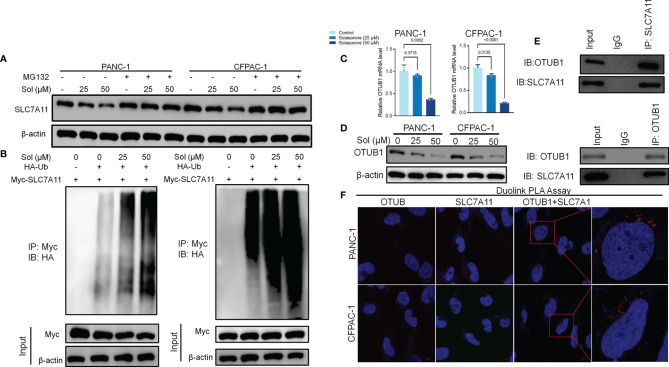
OTUB1-mediated Ub/proteasome pathway contributes to solasonine-mediated SLC7A11 degradation in pancreatic cancer cell. **(A)** Western blot demonstrating SLC7A11 protein levels in PANC-1 and CFPAC-1 cells after treatment with solasonine (25 or 50 μM) or MG132 (10 μM) for 6 h. **(B)** PANC-1 and CFPAC-1 cells were transiently transfected with the indicated constructs. Ubiquitinated SLC7A11 were immunoprecipitated and subjected to western blot analysis with the ubiquitin antibody. Prior to ubiquitination analysis, cells were treated with MG132. **(C, D)** qRT-PCR and Western blot demonstrating OTUB1 levels in PANC-1 and CFPAC-1 cells after treatment with solasonine for 24 h. **(E)** OTUB1 and SLC7A11 was determined in OTUB1 or SLC7A11-IP and total OTUB1 and SLC7A11 was determined on total extracts as input in PANC-1 cells. **(F)** Protein interactions were detected with Duolink^®^PLA labeled in red. Data are shown as mean ± SD for n=3.

**Figure 5:**
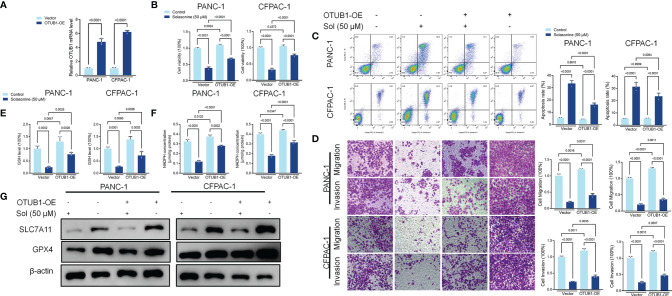
Solasonine suppressed OTUB1/SLC7A11 axis in PANC-1 and CFPAC-1 cells. **(A)** qRT-PCR demonstrating OTUB1 levels in PANC-1 and CFPAC-1 cells after transfection with OTUB1 overexpressing vector for 24 h. **(B)** CCK8 results showing the viability of PANC-1 and CFPAC-1 cells after treatment with solasonine or transfection with OTUB1-OE for 24 h. **(C)** Cell apoptosis, as detected by flow cytometry. **(D)** Cell migration and invasions were detected by Transwell assays. **(E, F)** Cellular GSH and NADPH levels were detected in PANC-1 and CFPAC-1 cells after treatment with solasonine or transfection with OTUB1-OE for 24 h. **(G)** Western blot demonstrating SLC7A11 and GPX4 levels in PANC-1 and CFPAC-1 cells after treatment with solasonine or transfection with OTUB1-OE for 24 h. Data are shown as mean ± SD for n=3.

We investigated the mechanisms through which solasonine negatively regulates SLC7A11 expressions while enhancing ferroptosis. It has been reported that SLC7A11 activates the proteasome-dependent degradation pathways (37, 38). PANC-1 and CFPAC-1 cells were co-treated with solasonine and the proteasome inhibitor, MG132. Co-incubation with MG132 alleviated solasonine-mediated SLC7A11 degradation (**Figure 4A**). Furthermore, solasonine accelerated SLC7A11 ubiquitination in a dose-dependent manner (**Figure 4B**). These findings suggest that solasonine induces protein degradation by promoting SLC7A11 ubiquitination. OTUB1, a deubiquitination enzyme, plays an essential role in regulating the stability of SLC7A11 and suppresses its ubiquitination (39). To verify the role of OTUB1 in pancreatic cancer, we extracted PAAD (Pancreatic adenocarcinoma) data from the TCGA database while normal tissue data were retrieved from the GTEx database. Then, expressions of OTUB1, SLC7A11 and GPX4 in tumor (n=179) and normal (n=171) tissues as well as their associations with survival outcomes were evaluated. Compared to normal tissues, mRNA levels of OTUB1, SLC7A11 and GPX4 were significantly upregulated in pancreatic cancer tissues (**Supplementary Figure 1A**). The area under the ROC curve for the TCGA cohort was 0.979 in GPX4, 0.776 in SLC7A11 and 0.974 in OTUB1. The predictive powers of OTUB1, SLC7A11 and GPX4 in pancreatic cancer were highly accurate (**Supplementary Figure 1B**). Kaplan-Meier overall survival curves, which were based on OTUB1, SLC7A11 and GPX4 levels from the TCGA PAAD database confirmed that patients with high expressions of OTUB1, SLC7A11 and GPX4 had significantly low survival outcomes, relative to those with low expressions (**Supplementary Figure 1C**). **Figures 4C, D** shows that after treatment with solasonine (25 and 50 μM), there were marked reductions in protein and mRNA levels of OTUB1 in PANC-1 and CFPAC-1 cells. Then, Co-Immunoprecipitation (CO-IP) assays showed that OTUB1 indeed interacts with SLC7A11 and highly sensitive Duolink^®^ *in situ* PLA assays were performed to reveal binding between SLC7A11 and OTUB1 in PANC-1 and CFPAC-1 cells (**Figures 4E, F**). Next, we transfected the OTUB1 overexpression plasmid (OTUB1-OE) into cells (**Figure 5A**). As shown in **Figures 5B, C**, overexpression of OTUB1 reversed the anti-proliferation and pro-apoptotic effects of solasonine on pancreatic cancer cells. Cell migration and invasion exhibited a similar pattern and were also promoted by overexpression of OTUB1 (**Figure 5D**). In addition, OTUB1-OE elevated GSH and NADPH concentrations as well as protein levels of SLC7A11 and GPX4 that had been suppressed by solasonine (**Figures 5E–G**). These results imply that solasonine-activated ferroptosis is accomplished by inhibition of OTUB1-mediated deubiquitination of SLC7A11.

### Solasonine Blocked TFAP2A to Positively Regulate OTUB1 Transcription

**Figure 6:**
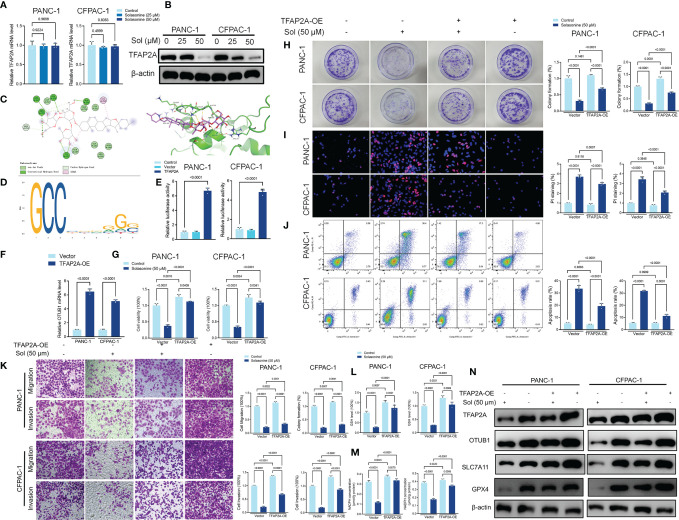
Solasonine suppressed TFAP2A levels in PANC-1 and CFPAC-1 cells. **(A, B)** qRT-PCR and Western blot for TFAP2A levels in PANC-1 and CFPAC-1 cells after treatment with solasonine for 24 h. **(C)** Theoretical binding mode of solasonine at the binding site of TFAP2A. **(D)** Sequence logo representing consensus TFAP2A binding motif from the JASPAR database (http://jaspar.genereg.net/). **(E)** Luciferase activities were detected in PANC-1 and CFPAC-1 cells after transfection with the TFAP2A-expressing vector. Luciferase reporter plasmid contained a 2 kb fragment upstream of the OTUB1 promoter. **(F)** qRT-PCR demonstrating TFAP2A levels in PANC-1 and CFPAC-1 cells after transfection with the TFAP2A overexpression vector for 24 h. **(G)** CCK8 results showing the viability of PANC-1 and CFPAC-1 cells after treatment with solasonine (50 μM) or transfection with TFAP2A overexpression vector for 24 h. **(H)** Cell migration and invasions were detected by Transwell assays after treatment with solasonine or transfection with TFAP2A-OE for 24 h. **(I)** PI staining (red) indicates apoptotic/necrotic cells. **(J)** Cell apoptosis detected by flow cytometry after treatment with solasonine or transfection with TFAP2A-OE for 24 h. **(K)** Cell migration and invasions were detected by Transwell assays. **(L, M)** Cellular GSH and NADPH levels in PANC-1 and CFPAC-1 cells after treatment with solasonine or transfection with TFAP2A-OE for 24 h. **(N)** Western blot demonstrating TFAP2A, OTUB1, SLC7A11 and GPX4 levels in PANC-1 and CFPAC-1 cells after treatment with solasonine or transfection with TFAP2A-OE for 24 h. Data are shown as mean ± SD for n=3.

Transcription factor activating enhancer binding protein 2 alpha (TFAP2A) regulates the transcription of downstream genes by binding to CG-rich sequences through the DNA-binding domain within the COOH- terminus (40). TFAP2A has been found to be associated with a variety of tumors and is highly expressed in a variety of tumor cells including glioma, gastric cancer, bile duct cancer, breast cancer, and colorectal cancer, regulating tumor proliferation and apoptosis (41–44). To clarify the mechanism of TFAP2A in pancreatic cancer, we first analyzed the TCGA and GTEx databases and compared the expressions as well as survival differences of TFAP2A in tumors (n=179) and normal tissues (n=171). The mRNA levels of TFAP2A in pancreatic cancer tissues were significantly upregulated, compared to normal tissues (**Supplementary Figure 2A**). The area under ROC curve of the TCGA cohort was 0.976 in TFAP2A, implying a highly accurate predictive power of TFAP2A in pancreatic cancer (**Supplementary Figure 2B**). Kaplan-Meier overall survival curves that were based on TFAP2A levels from the TCGA PAAD database confirmed that patients with high expressions of TFAP2A had significantly low survival outcomes (**Supplementary Figure 2C**). Solasonine suppressed TFAP2A protein levels, however, it did not alter its mRNA levels (**Figures 6A, B**). Molecular Docking revealed that solasonine formed hydrogen bonds with Lys257, Arg255, ala256 and gly250 of TFAP2A (**Figure 6C**). Interestingly, using JASPAR (http://jaspar.genereg.net), we found that there is a TFAP2A binding site in the promoter region of OTUB1 (**Figure 6D**). Luciferase assays showed that TFAP2A transcriptionally activates the OTUB1 promoter region (**Figure 6E**). Besides, overexpression of TFAP2A (TFAP2A-OE) increased the mRNA levels of OTUB1 (**Figure 6F**). We examined the correlations among TFAP2A, OTUB1, SLC7A11 and GPX4 from the TCGA database. As shown in **Supplementary Figures 2D, E**, there were significantly positive correlations among TFAP2A, OTUB1, SLC7A11 and GPX4. Furthermore, a nomogram was plotted for the TCGA cohort to calculate the risk score and predict 1-, 3-, and 5-year OS of pancreatic cancer patients (**Supplementary Figure 3**). **Figures 6G–K** shows that TFAP2A-OE inhibits the effects of solasonine on pancreatic cancer cells to inhibit their proliferation, metastasis and to promote their apoptosis. Besides, TFAP2A-OE elevated GSH and NADPH concentrations, which had been suppressed by solasonine (**Figures 6L, M**) and activated the protein expressions of OTUB1 and its downstream SLC7A11 as well as GPX4 in PANC-1 and CFPAC-1 cells (**Figure 6N**).

### Solasonine Suppressed Pancreatic Cancer Cell Growth and Metastasis *In Vivo*

**Figure 7:**
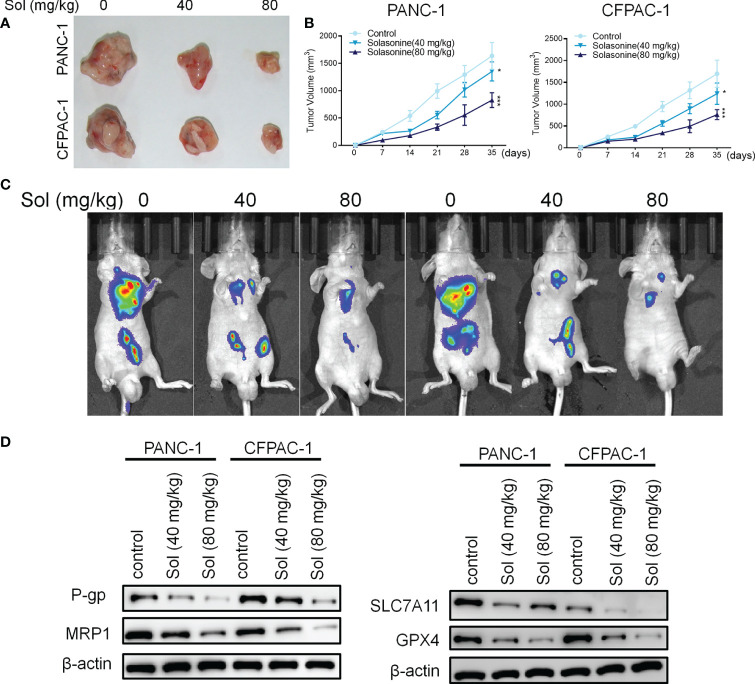
Solasonine suppressed pancreatic cancer *in vivo*. **(A)** Representative images of xenograft tumors isolated from nude mice in different groups. **(B)** Tumor sizes in the different groups. **(C)** Live imaging showing metastasis of PANC-1 and CFPAC-1 cells after intravenous tail injection with solasonine in different weeks in the pancreatic cancer metastasis model. **(D)** Western blot showing TFAP2A, OTUB1, SLC7A11 and GPX4 levels in xenograft tumors. n=6, data are shown as mean ± SD.

Mice xenograft models of PANC-1 and CFPAC-1 cells confirmed that solasonine (40 and 80 mg/kg) treatment suppressed tumor volumes, compared to the control group (**Figures 7A, B**). Next, we constructed a pancreatic cancer metastasis model. Tumor metastasis in mice was evaluated by a live fluorescence imaging technique. Live imaging showed that solasonine (40 and 80 mg/kg) abrogated NSCLC metastasis, compared to the control group (**Figure 7C**). Besides, solasonine suppressed protein levels of drug resistance related proteins (MRP1 and P-gp) as well as those of ferroptosis related proteins (SLC7A11 and GPX4) (**Figure 7D**). These results indicate solasonine inhibited pancreatic cancer cell growth and metastasis *in vivo.*

## Discussion

Pancreatic cancer is one of the most malignant cancers of the gastrointestinal tract. It is highly aggressive, with a short time course, rapidly progressive and metastatic, and is associated with a very low survival rate (45). Treatment of pancreatic cancer is very challenging, probably due to its heterogeneity and susceptibility to drug resistance. It poorly responds to first-line treatment, such as gemcitabine (46). Therefore, it is necessary to develop new low-toxic and highly effective anti-pancreatic cancer drugs. Solasonine, as the main active ingredient of the Chinese herbal medicine, *Solanum nigrum L.*, has shown significant inhibitory proliferative effects on various tumor cells, however, its effects in pancreatic cancer have not been fully established.

Transcription factors (TFs) regulate gene expressions by binding specific DNA sequences. They are involved in tumor cell processes, such as proliferation, apoptosis and migration. TFAP2A, a member of the AP-2 family of TFs, is expressed early in embryogenesis and it regulates the transcription of neural as well as epithelial genes. Aberrant expressions of TFAP2A have been reported in various cancers (47). The TFAP2A and TFAP2G transcription factors are involved in regulation of proliferation, differentiation as well as apoptosis in normal breast epithelium and breast cancer. In invasive breast cancer, TFAP2A levels were established to be lower, compared to normal breast and ductal carcinoma *in situ*. These findings imply that in breast cancer, TFAP2A is a tumor suppressor (48). In human bladder cancer cells, TFAP2A was established to be positively correlated with TP63, and it positively regulated TP63 expressions (49). We found that TFAP2A expressions were significantly elevated in pancreatic cancer tissues and were associated with poor prognostic outcomes. *In vitro*, overexpressed TFAP2A enhanced pancreatic cancer cell proliferation, suppressed their apoptosis, and promoted the expressions of downstream OTUB1. Notably, it has been reported that TFAP2A regulates iron death in gallbladder cancer cells by activating the NRF2 pathway. NADPH levels in gallbladder cancer cells were significantly reduced after inhibition of TFAP2A (50). This result is comparable to ours. However, our results only demonstrate that TFAP2A transcriptionally regulates OTUB1, but do not clarify the specific promoter region for TFAP2A in OTUB1. In previous studies, we analyzed DNA sequences of the OTUB1 promoter and identified E-boxes, which are putative DNA-binding sites for TFAP2A.

The ubiquitin-protease system consists of ubiquitin, ubiquitin activating enzyme (E1), ubiquitin-conjugating enzyme (E2), ubiquitin ligase enzyme (E3), deubiquitinase (DUB) and proteasome (51). Deubiquitinating enzymes specifically sever the peptide bond between the substrate protein and ubiquitin or block the transmission of ubiquitin by ubiquitinating enzymes, leading to reversal of the ubiquitination process and self-regulation of the body’s protein levels to maintain homeostasis of the intracellular environment (52). The ubiquitination process can result in three different types of ubiquitination, including mono-ubiquitination, where a single site is ubiquitinated, poly-mono-ubiquitination, where multiple sites are ubiquitinated, and poly-ubiquitination, where a single lysine site forms a poly-ubiquitin chain (53). As an important post-translational modification process of proteins, ubiquitination plays an important function *in vivo*. It also plays an important role in the development of malignant tumors (54). More than 90 deubiquitinating enzymes have been identified, including OTUB1 (55). The deubiquitinating enzyme, OTUB1, has the ability to target various substrate proteins for degradation, many of which are associated with tumor development. OTUB1 can target the ubiquitination of c-IAP-l, which is involved in regulation of NF-kB and MAPK signaling pathways as well as TNF-dependent cell death. Knocking down OTUB1 downregulates the stability of the c-IAP-l protein and induces apoptosis (56). OTUB1 can also inhibit the ubiquitination of FOXM1, stabilize FOXM1 protein levels, downregulate FOXM1 protein levels in breast cancer, and increase the sensitivity of breast cancer cells to chemotherapeutic agents (57). In addition, OTUB1 can inhibit the monoubiquitylation of RAS, promote lung cancer progression (58), stabilize Snail protein levels, and promote the metastasis of esophageal squamous carcinoma (59). Liu et al. (39) found that OTUB1 directly interacts with and stabilizes SLC7A11, and that inactivation of OTUB1 destabilized SLC7A11, leading to inhibition of tumor xenograft growth in mice, which was associated with reduced activation of ferroptosis. Therefore, in various malignant tumors, OTUB1 acts as a pro-oncogene and promotes tumor progression. Analysis of the TCGA database confirmed that OTUB1 expression was significantly elevated in pancreatic cancer tissues than in normal tissues, and that patients with high OTUB1 expressions in pancreatic cancer have significantly lower survival rates than those with low expressions. *In vitro*, solasonine dose-dependently inhibited OTUB1 mRNA and protein expressions in PANC-1 and CFPAC-1 cells. Besides, CO-IP and Duolink^®^ PLA assays confirmed the interactions between OTUB1 and SLC7A11 and the deubiquitinating effects of OTUB1 on SLC7A11 in PANC-1 and CFPAC-1 cells.

As a form of cell death that is associated with iron-dependent peroxidation of polyunsaturated phospholipids in cell membranes, ferroptosis is generally induced through two pathways. One is through depletion of GSH and cysteine or inhibition of the phospholipid hydroperoxidase, GPX4, while the other is by increasing intracellular ferrous ion levels (60). A previous study revealed that ADP Ribosylation Factor 6 endowed pancreatic cancer cells to a status that is sensitive to RSL3-induced lipid peroxidation and regulated gemcitabine resistance (61). Besides, bioinformatics analysis showed that ferroptosis is strongly associated with CD8+ T cells, type II interferon responses and immune checkpoints in pancreatic cancer, suggesting that immunotherapy and chemotherapy combined with ferroptosis inducers are viable treatment options for pancreatic cancer (62). Or findings show that solasonine induces pancreatic cancer cell death by causing lipid peroxidation. When the increased ferrous ions were chelated by DFO or further increased by FAC, lipid peroxidation was inhibited or enhanced accordingly. Inhibition of lipid peroxidation with Fer-1 significantly rescued solasonine-induced pancreatic cancer cell death. These findings are consistent with those of a previous study (60). Thus, solasonine-induced ferroptosis occurs in pancreatic cancer cells. Many studies have proposed that ferroptosis is closely related to tumor drug resistance. On the one hand, iron overload in tumor cells catalyzes ROS production, which to some extent can satisfy their proliferation needs; on the other hand, when tumor cells are exposed to chemotherapeutic drugs, a large amount of ROS production is induced, and excessive accumulation of ROS poses a great challenge to the survival of tumor cells. Once tumor cells initiate some mechanism to alter their metabolic microenvironment and inhibit ROS formation, drug resistance is induced (63). Sato et al. (64) showed a good synergistic effect after pretreatment of cisplatin-resistant ovarian cancer cells with the ferroptosis agonist, Erastin, which increased their sensitivity to cisplatin. In addition, Roh et al. (65) found that the ferroptosis agonist, Erastin reversed cisplatin resistance in head and neck tumors. Solasonine has also been reported to have the effect of inhibiting p-gp protein expression in tumor cells and reversing drug resistance (66, 67). Our results found that the expression of drug resistance-associated proteins MRP1 and P-gp was significantly reduced after intervention with solasonine in pancreatic cancer cells, while the expression of MRP1 and P-gp was reversed after intervention with Fer-1, an iron death inhibitor. This also confirmed that solasonine to inhibit drug resistance protein expression in pancreatic cancer cells by activating iron death. In future studies, we will construct drug-resistant pancreatic cancer cell lines using first-line chemotherapeutic agents such as gemcitabine and 5-fu and intervene with solasonine to further clarify the mechanism of drug resistance inhibition.

In conclusion, solasonine is involved in ferroptosis by suppressing TFAP2A-mediated transcriptional upregulation of OTUB1, thereby activating ubiquitinated degradation of SLC7A11 and promoting pancreatic cancer cell ferroptosis. These results form the basis for the potential application of solasonine as a small-molecule ferroptosis agonist to facilitate further developments of anticancer therapies.

## Data Availability Statement

The raw data supporting the conclusions of this article will be made available by the authors, without undue reservation.

## Ethics Statement

All animal experiments were conducted in accordance with recommendations of the Animal Care Committee of Shanghai University of Traditional Chinese Medicine. The protocol was reviewed and approved by the Shanghai University of Traditional Chinese Medicine Institutional Review Board (Permit Number: PZSHUTCM210305012). Surgery was performed under sodium pentobarbital anesthesia with all efforts being made to minimize suffering.

## Author Contributions

JZ and XL designed experiments. XL, CH, MH, CL, and XS performed experiments. XL and KY analyzed the results. XL and CH wrote the manuscript. HG and JZ revised and approved the submitted version.

## Funding

This work was financially supported by National Natural Science Foundation of China (No: 81403305) and Shanghai Municipal Commission of Health and Family Planning (No: 2018JP012).

## Conflict of Interest

The authors declare that the research was conducted in the absence of any commercial or financial relationships that could be construed as a potential conflict of interest.

## Publisher’s Note

All claims expressed in this article are solely those of the authors and do not necessarily represent those of their affiliated organizations, or those of the publisher, the editors and the reviewers. Any product that may be evaluated in this article, or claim that may be made by its manufacturer, is not guaranteed or endorsed by the publisher.
