# Supplementary material for: Solasonine Inhibits Pancreatic Cancer Progression With Involvement of Ferroptosis Induction
Source: Front Oncol. 2022 Apr 12;12:834729. doi: 10.3389/fonc.2022.834729 (PMC9039314; doi:10.3389/fonc.2022.834729)
Supplement: Supplementary file 1 [file DataSheet_1.docx]

**Supplementary Figure 1:** A. GPX4, SLC7A11 and OTUB1 levels in PAAD (Pancreatic adenocarcinoma) tumor tissues in the TCGA and GTEx databases. B. ROC curves for GPX4, SLC7A11 and OTUB1 levels in PAAD patients in the TCGA and GTEx databases. C. Overall survival outcomes of PAAD patients in relation to GPX4, SLC7A11 and OTUB1 levels in the TCGA database. ***p＜0.001.

**Supplementary Figure 2:** A. TFAP2A levels in PAAD tumor tissues in the TCGA and GTEx databases. B. ROC curves for TFAP2A expressions in PAAD patients in the TCGA and GTEx databases. C. Overall survival of PAAD patients in relation to TFAP2A expressions in the TCGA database. D. Spearman correlation analysis was used to determine the correlations between GPX4, SLC7A11, OTUB1 and TFAP2A in PAAD in the TCGA database. E. GPX4, SLC7A11 and OTUB1 expression heat map and correlations with TFAP2A co-expressed genes. ***p＜0.001.

**Supplementary Figure 3:** The nomogram combining TFAP2A-based risk values with several clinical factors accurately predicted the survival outcomes of patients with PAAD.

**Methods**

**Total RNA isolation and RT-qPCR assay**

Total RNA was isolated using TRIzol (Invitrogen). Primers for OTUB1 were attained from GenePharma (Shanghai, China). *β-actin* was used as the reference gene for mRNAs. Gene expression was quantified using the 2^−ΔΔCt^ method as former describe[[1](#_ENREF_1)].

**Protein extraction and western blot analysis**

Cytosolic and nuclear proteins in cells or tissues were isolated as described in NE-PER™ Nuclear and Cytoplasmic Extraction Reagents. and protein concentration was determined using a BCA protein assay kit. Approximately 30 μg of protein from each sample was separated by 10% sodium dodecyl sulphate-polyacrylamide gel electrophoresis (SDS-PAGE) and transferred to a polyvinylidene fluoride (PVDF) membrane. Membranes were blocked with 5% skimmed milk in TBST and incubated with primary antibodies overnight at 4°C [[1](#_ENREF_1)]. Primary antibodies were obtained from Abcam: TFAP2A (ab236043), OTUB1 (ab283705), E-Cadherin (ab40772), N-Cadherin (ab18203), SLC7A11 (ab175186), GPX4 (ab231174) and β-actin (ab8226). All dilutions were 1:1,000 except β-actin is 1:3000.

**Cell viability and clonability assays**

According to the manufacturer’s instructions, the cell counting kit-8 (CCK-8) system (Dojindo, Japan) was used to measure the activity of transfected cells inoculated into 96-well plates at a density of 1 × 10^4^ cells/Wells. In short, before incubating the plate for 1 h at 37 °C in the dark, add 10 m of CCK-8 solution to each well, and measure the absorbance of each well at 450 nm with the microplate reader (Tecan, Switzerland). In the colony formation assay, cells were seeded with low density (1000 cells/plate) and cultured until visible clones appeared, then stained with Giemsa and counted the number of colonies.

**Migration and invasion assays**

In the Transwell migration assays and the invasion assays, the cells were first placed in serum-free medium, and the medium supplemented with 10% serum was placed in the lower chamber as a chemoattractant. The former was seeded with 1 × 10^4^ cells in an upper chamber with a non-coated membrane (24-well insert; 8 mm pore size; BD Biosciences), and the latter seeded with 2 × 10^5^ cells with a Matrigel-coated membrane (24-well insert; 8 mm pore size; BD Biosciences). The cells were incubated in a tissue culture incubator at 37 °C and 5% CO_2_ for 16 h, after which the ummigrated/non-invasive cells on the upper sides of the Transwell membrane filter insert were gently wiped off with a cotton swab. On the underside of the insert, cells were stained with crystal violet and counted.

**Apoptosis assay**

Annexin V/PI staining was performed to assess cell apoptosis. In brief, cells (1 × 10^3^) were seeded into the wells of 96-well plates and exposed to Solasonine (50 μM) for one day, then trypsinized, washed twice with cold phosphate-buffered saline (PBS), resuspended in binding buffer (500 μL), and finally stained with annexin V-fluorescein isothiocyanate conjugate (5 μL) and PI (5 μL) at room temperature for 15 min in the dark. The proportion of apoptotic cells was determined by flow cytometry. Each experiment repeats three times.

**Measurement of GSH and NADPH**

Commercial assay kits (NADPH: S0179, Beyotime Biotechnology; GSH: A061-1, NanJing JianCheng Bioengineering) were used to measure the concentrations of glutathione (GSH), and nicotinamide adenine dinucleotide phosphate (NADPH) in hippocampal tissue according to manufacturers’ instructions, respectively.

**Metabolomics data collection and analysis**

Cells treated with or without Solasonine (50 μM) were used for metabolomics analysis. The cells were seeded into the wells of 6-well plates at an initial density of 106/well. After an appropriate culture period, the supernatant was discarded and each well was washed three times with PBS. Then, 2 mL of cold (4 °C) methanol were added to each well and adhered cells were scraped free and lysed with a cell pulverizer to fully extract the metabolites. Finally, the supernatant was centrifuged at 14,000 × g for 10 min before liquid chromatography-mass spectrometry analysis. All resulting data were processed with Compound Discoverer 2.1 software (Thermo Fisher Scientific). Orthogonal partial least squares discriminant analysis (OPLS-DA) and principal component analysis were conducted using SMICA-P 14.0 software (MKS Umetrics AB, Umeå, Sweden). Metabolite identification was based on product ion spectra and accurate masses. Pathway analysis was performed with MetaboAnalyst 4.0 metabolomics software.

**Immunoprecipitation (IP)**

For OTUB1 or SLC7A11 immunoprecipitation, cells were lysed in SDS-containing buffer, boiled, and diluted 20-fold in the above NP-40 lysis buffer. After centrifugation, the supernatant was incubated with OTUB1 or SLC7A11 antibody at 4 °C overnight. Followed by washing with the NP-40 lysis 5 times, the beads were incubated with protein A/G agarose at 4 °C for 4 h. Finally, the beads were washed in the NP-40 lysis buffer ﬁve times, boiled and analyzed by western blot.

**Proximity ligation assay (PLA)**

Cells were prepared in 8-well chamber slides and allowed to attach for 24 hours. Cells were then rinsed in PBS, fixed in 100% methanol for 20 min, washed three times with PBS, and incubated overnight with protein-specific antibodies at 4°C. Proximity ligation assays were performed using the Duolink InSitu PLA Assay Kit (Sigma-Aldrich), and nuclei were DAPI stained. Specimens were mounted using Vectashield mounting media and analyzed under a LSM 710 confocal microscope (Carl Zeiss). Numbers of in situ PLA signals per cell were counted by semiautomatic image analysis using Blob Finder V3.0.

**Molecular Docking Analysis**

Molecular docking analyses were performed to investigate interactions between Solasonine and TFAP2A using AutoDock Vina 1.1.2 [[2](#_ENREF_2)]. A 3D structure of Solasonine was drawn using ChemBioDraw Ultra 14.0 and converted to a 3D structure by ChemBio 3D Ultra 14.0. The homology model was obtained from SWISS-MODEL (https://www.swissmodel.expasy.org/), and ligand binding sites of the proteins were predicted by POCASA 1.1 in which the principle was consistent with AutoDock software due to the lack of TFAP2A protein structure in the PDB database. AutoDockTools version 1.5.6 [[2](#_ENREF_2), [3](#_ENREF_3)] was employed to generate docking input files. The crystallographic ligands were extracted and fed into a docking database for redocking, and hydrogen atoms added. An auxiliary program AutoGrid was used to generate a docking area that was defined as a 40 × 40 × 40 3D grid centered on the ligand binding site with a 0.375 Å grid space. All bond rotations for the ligands were ignored in this study. The best scoring pose from Vina docking score evaluations was selected for further analyses using PyMoL 1.7.6 software.

**Reference**

[1] Zhang X, Hu F, Li C, Zheng X, Zhang B, Wang H, et al. OCT4&SOX2-specific cytotoxic T lymphocytes plus programmed cell death protein 1 inhibitor presented with synergistic effect on killing lung cancer stem-like cells in vitro and treating drug-resistant lung cancer mice in vivo. Journal of cellular physiology. 2019;234:6758-68.

[2] Trott O, Olson AJ. AutoDock Vina: improving the speed and accuracy of docking with a new scoring function, efficient optimization, and multithreading. Journal of computational chemistry. 2010;31:455-61.

[3] Sanner MF. Python: a programming language for software integration and development. Journal of molecular graphics & modelling. 1999;17:57-61.
