# Supplementary figures and images for: Solasonine Inhibits Pancreatic Cancer Progression With Involvement of Ferroptosis Induction
Source: Front Oncol. 2022 Apr 12;12:834729. doi: 10.3389/fonc.2022.834729 (PMC9039314; doi:10.3389/fonc.2022.834729)

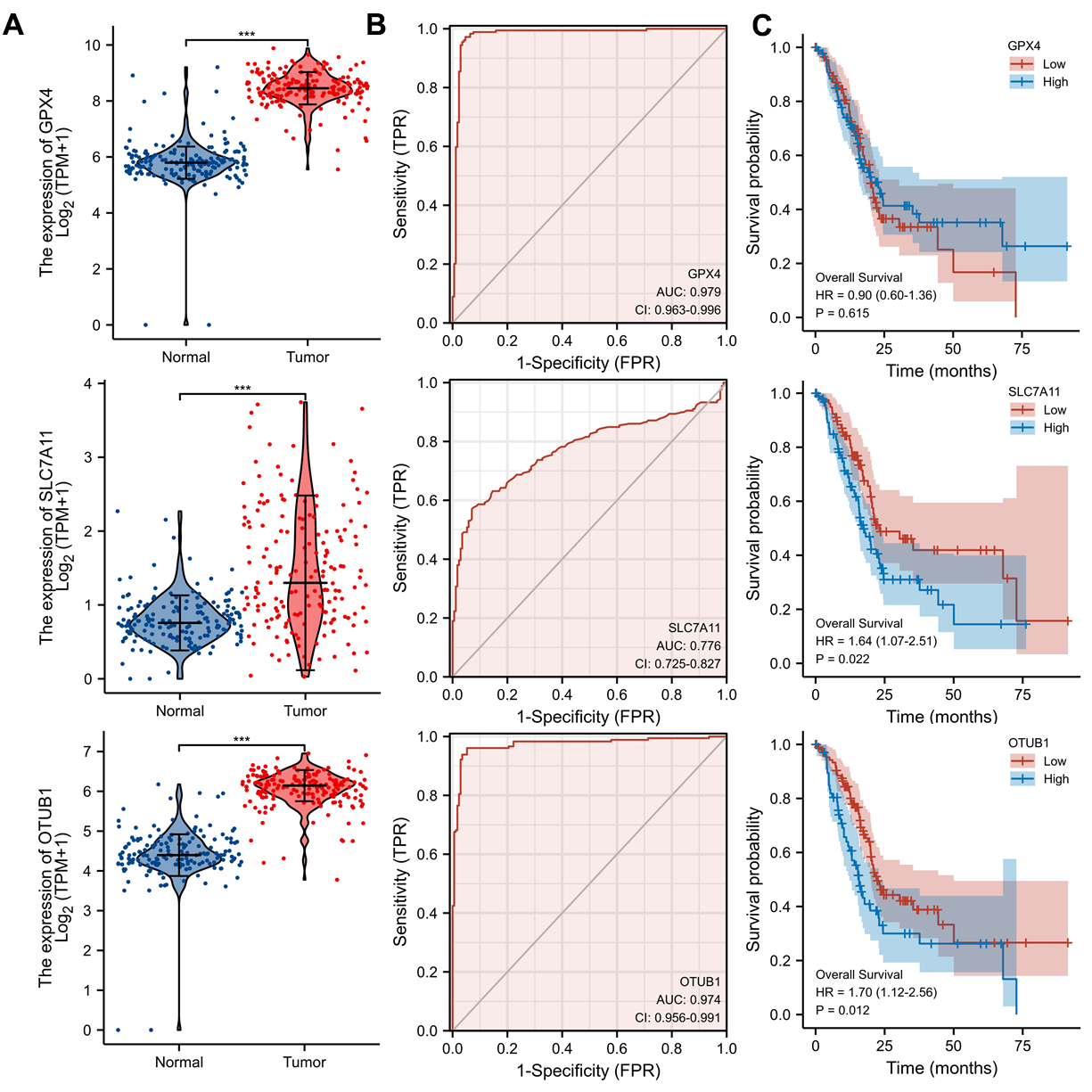

Supplement: Supplementary file 2 [file Image_1.tif]

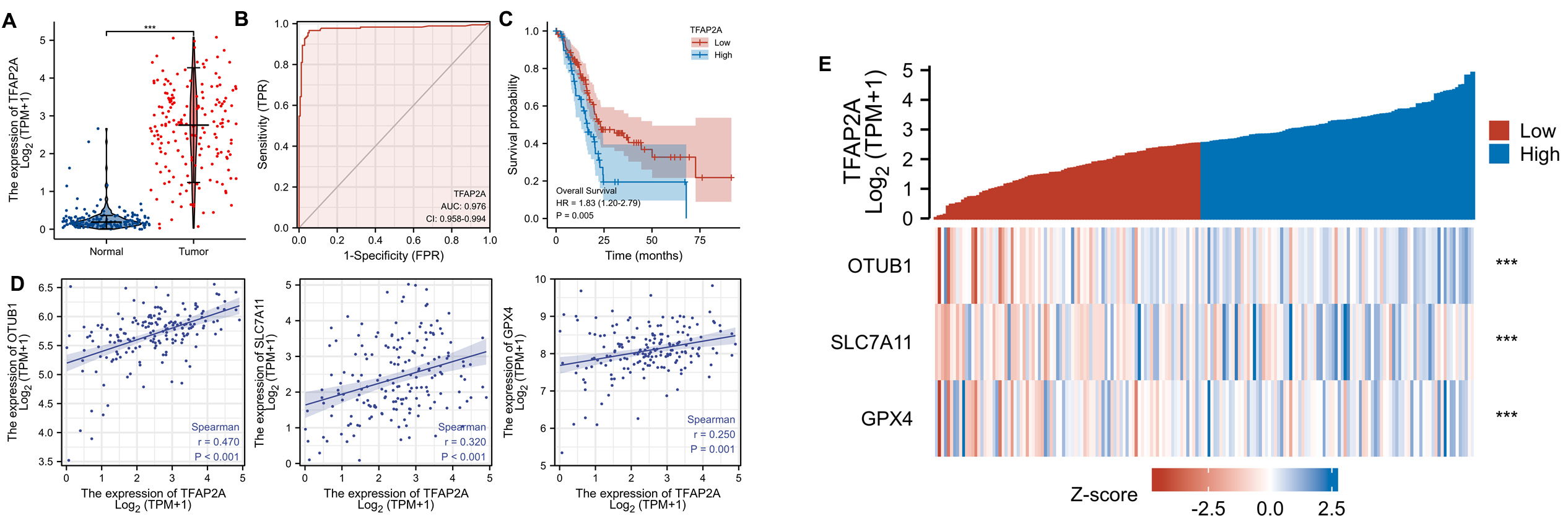

Supplement: Supplementary file 3 [file Image_2.tif]

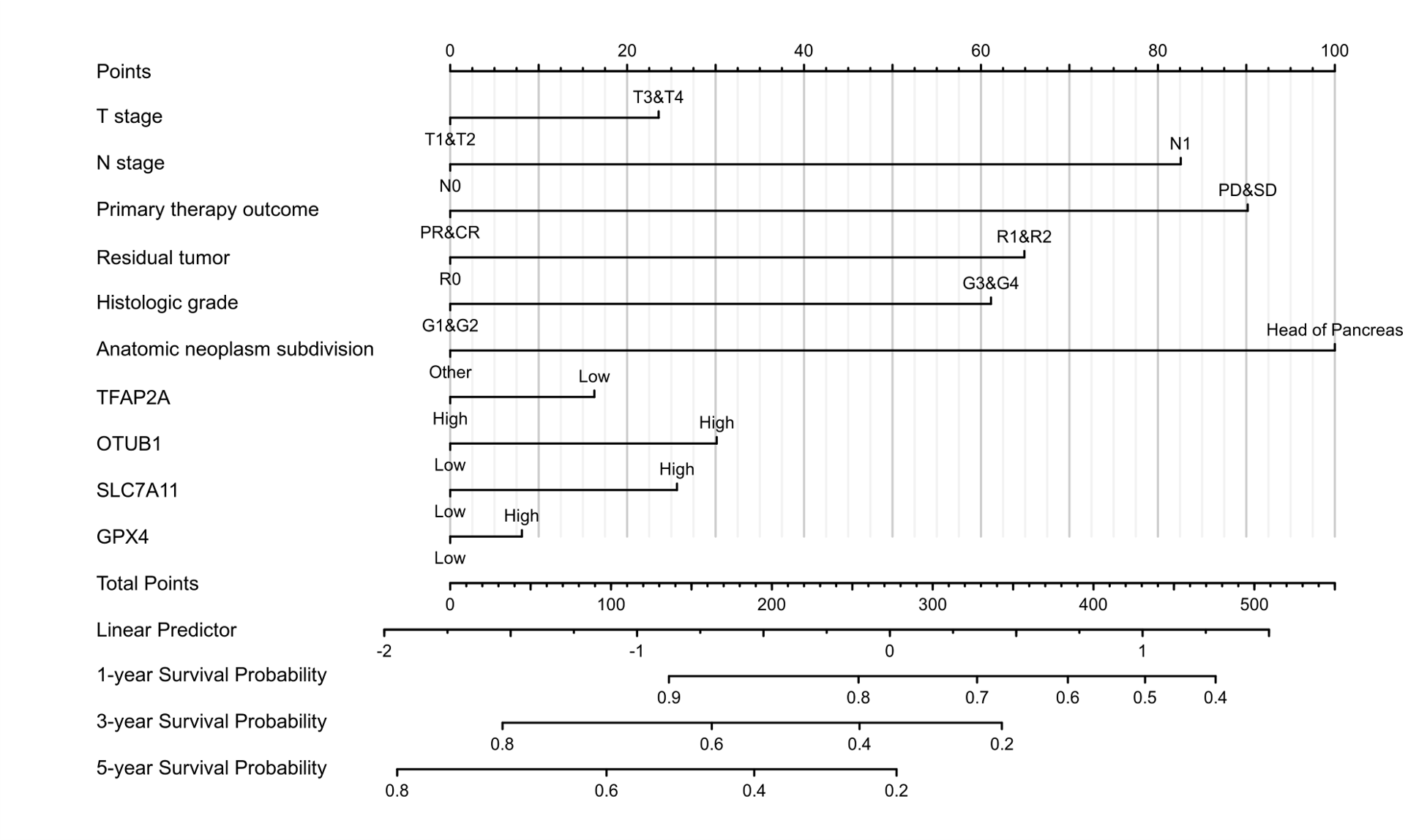

Supplement: Supplementary file 4 [file Image_3.tif]
